# Maternal Immune Characteristics at Mid and Late Pregnancy Are Mostly Independent of Fetal Allogeneity in Mice

**DOI:** 10.1111/aji.70089

**Published:** 2025-06-01

**Authors:** Anne Laskewitz, Lieske Wekema, Marijke M. Faas, Jelmer R. Prins

**Affiliations:** ^1^ Division of Medical Biology Department of Pathology and Medical Biology University Medical Center Groningen University of Groningen Groningen the Netherlands; ^2^ Department of Obstetrics and Gynaecology University Medical Center Groningen University of Groningen Groningen the Netherlands

**Keywords:** allogeneity, immune adaptations, maternal–fetal interactions, murine model, pregnancy immunology

## Abstract

**Problem:**

During pregnancy, the maternal immune system undergoes different adaptations to establish tolerance toward the fetus. Several factors are known to affect maternal immune adaptations, including fetal allo‐antigens. Although, the effects of fetal antigens have clearly been shown in early pregnancy, the influence of these antigens in mid‐ and late pregnancy are not fully known.

**Method of Study:**

In this study, we investigated the impact of fetal allo‐antigens during pregnancy on maternal immune adaptations by comparing immunological changes in semi‐allogeneic and syngeneic pregnancies in mice. Pregnant mice were analyzed at gestation day 10 (GD10) and gestation day 18 (GD18), and immune responses were assessed in the spleen, uterus draining para‐aortic lymph nodes (PALN) and peripheral blood analyzing T cells, monocytes and their subsets. In addition, T cells were analyzed after stimulation using PMA or male antigens.

**Results:**

Analyses of memory T cells, Tregs and monocytes (and subsets) demonstrated that fetal semi‐allogeneity only had a minor influence on the maternal immune parameters in mid and late gestation. Immune stimulation experiments, using either PMA or syngeneic or allogeneic paternal antigens, revealed minor differences in cytokine production between general immune stimulation and paternal specific stimulation.

**Conclusions:**

Our results show that immune adaptations ad mid and late pregnancy are likely not mainly driven by fetal allo‐antigens. This study contributes to a deeper understanding of the maternal immune system during pregnancy (either syngeneic or semi‐allogeneic).

## 1 Introduction

During pregnancy, the maternal immune system undergoes modifications to establish tolerance toward the fetus, which expresses both maternal and paternal antigens [1, 2]. Therefore, different maternal immune adaptations take place during pregnancy, which encompass both the innate and the adaptive immune system.

Within the adaptive immune system the role and dynamics of T cells during pregnancy are mostly studied. T cells are divided into CD4^+^ and CD8^+^ cells, and CD4^+^ T cells can be further divided into various subsets, among which Th1, Th2, memory T cell, and Tregs are the most extensively studied in the context of pregnancy. Th1 are important in early pregnancy, during blastocyst implantation, but may be detrimental to pregnancy later on, since they induce immune responses to foreign cells [3]. Both Th2 cells and Treg cells are important in regulating the immune response against paternal antigens and are often increased in pregnancy [4, 5, 6]. At the end of pregnancy, cytotoxic T cells, are known to recognize paternal‐fetal antigens [7, 8]. Memory T cells are able to recognize foreign antigens and are able to elicit a stronger immune response upon encountering the same antigen [9]. During pregnancy, memory T cells have different phenotypes and functions compared to memory T cells in non‐pregnant individuals [2, 10]. CD4^+^ Treg memory cells, which are mostly studied in pregnancy, show fetal antigen specificity and are important for reproductive success in subsequent pregnancies [10].

Another part of the immune system, the innate immune system, forms the first line of defense against invading pathogens [11]. The innate immune system consists of different immune cells. One of the important antigen‐presenting cells and mediators of the immune response is monocytes. Monocytes can be divided into three subtypes: classical, intermediate, and non‐classical monocytes [11]. During pregnancy, there is increased activation of monocytes and an increase in non‐classical monocytes, together with a decrease of classical monocytes in both humans and rodents [12, 13]. This suggests a proinflammatory condition during pregnancy.

Several factors are known to affect maternal immune adaptations during pregnancy. These include hormones, like progesterone and estrogen, which have been shown to influence the maternal immune subsets [14, 15]. In addition, various factors released by the placenta, such as cytokines, exosomes or microparticles are also known to affect the maternal immune system [16, 17]. More recently, studies have indicated that also the maternal gut microbiome is an important factor in adapting the maternal immune system to pregnancy [18, 19]. Different immune adaptation processes take place at varying stages throughout pregnancy. At gestational day (GD) 10, which corresponds to mid‐gestation in mice, the process of spiral artery remodeling takes place. During this process, vascular endothelial cells are replaced with trophoblast cells [20]. Previous research indicates that from this day onwards, paternal‐specific memory cells are found in the para‐aortic lymphn nodes (PALN), suggesting that potential effects of fetal allo‐antigens would be apparent from this day onward [10]. Since the number of paternal‐specific cells are increasing during gestation and since GD18 of mice pregnancy is almost the end of pregnancy, the effect of fetal allogeneity is likely to be most pronounced at GD18 [10]. However, the specific effects of fetal allo‐antigens in adapting immune cell subsets in mid and late pregnancy are relatively unknown.

In the present study, we evaluated the effects of the presence of fetal allo‐antigens on maternal immune cell subsets during mid and late pregnancy in mice. We mated Balb/cJ female mice with either Balb/cJ male mice (syngeneic pregnancy) or with C57BL/6 male mice (semi‐allogeneic pregnancy). We analyzed subsets and function of T cells and monocytes at GD10 and GD18 in the uterus draining para‐aortic lymph nodes (PALN), spleen and peripheral blood.

## 2 Material and Methods

### 2.1 Experimental Design

All mice experiments were approved and performed in accordance with the guidelines and approval of the Central Committee for Animal Experimentation in The Netherlands (AVD1050020185904). Balb/cJ female mice, Balb/cJ male mice, and C57BL/6 male mice were purchased from Jackson Laboratories (ME, USA) and were 3–6 months of age when used in the experiment. All female mice were co‐housed, with a maximum of five mice per cage, in individually ventilated cages. Mice had access to a standard diet and sterile water and there was a regulated 12/12 h light schedule. Vaginal smears were taken to determine the phase of the estrus cycle, when in the pro‐estrus phase, females were randomly co‐housed with either a Balb/cJ male (syngeneic pregnancy) or a C57BL/6 male (semi‐allogeneic pregnancy). The next day, mice were separated and this was considered gestational day 0 (GD0). Pregnant mice were sacrificed at GD10 or GD18. For both time points (GD10 and GD18), 10 mice from syngeneic pregnancies and semi‐allogeneic pregnancies were sacrificed. Since both experiments (GD10 and GD18) were performed at different timepoints, in order to prevent a potential time bias we included a non‐pregnant control group in both experiments and refrained from directly comparing the groups.

Mice were anesthetized with isoflurane/O_2_ and a cardiac puncture was used to collect blood into EDTA tubes (BD‐Plymouth, UK). Hereafter, the para‐aortic lymph nodes (PALN) and spleens were collected. All tissues were stored on ice and processed immediately after collection.

### 2.2 Cell Isolation

To obtain a cell suspension from the spleen and PALN, organs were crushed between object glasses and resuspended in 3 mL (spleen) or 2 mL (PALN) RPMI containing 10% heat inactivated fetal calf serum (dFCS). To lyse red blood cells from the spleen cell suspensions, 4 mL cold Ammonium chloride was added and incubated for 10 min on ice. Hereafter, the suspensions were washed twice with 10% FACS buffer (phosphate buffered saline (PBS) + 2% dFCS), and resuspended in 2 mL 10% FACS buffer. Cell suspensions of spleen and PALN were filtered using a 35 µm filter (Corning, the Netherlands). Cells were counted with the Coulter counter (Beckman Coulter Life Sciences, USA). Splenocytes were frozen for later use in the stimulation assays. To allow for freezing, freezing medium, containing dFCS and 10% DMSO, was added after which the cells were slowly frozen in liquid nitrogen.

### 2.3 Cell Staining

T‐cell staining: 1 × 10^6^ cells spleen or PALN cells were added to a 96‐well plate and centrifuged for 3 min. To allow for live/dead staining, cells were incubated with Zombie RED for 15 min (Bioleged, USA), and two washing steps with FACS buffer were performed. Hereafter, cells were incubated with 50 µL extracellular blocking medium containing 1% (v/v) FC block (eBioscience) for 10 min. Cells were centrifuged and supernatant was discarded, then cells were resuspended in 25 µL extracellular antibody mix (Table S1) for 30 min, followed by two washing steps with FACS buffer after which cells were resuspended in Fixation/Permeabilization buffer (BD Biosciences, USA) and stored at 4°C for a maximum of 16 h. After incubation in the fixation/permeabilization buffer, cells were washed twice with perm/wash buffer (BD Biosciences, USA) and the intracellular antibody mix was added (Table S1) for 30 min. Hereafter, cells were washed once with perm/wash buffer (BD Biosciences, USA), and then once with dPBS. Cells were resuspended in dPBS and stored at 4°C for a maximum of 2 h before acquiring.

Monocyte staining: Whole blood was diluted in a 1:1 ratio with RPMI, and 200 µL diluted blood was used for staining. The diluted blood was incubated with 50 µL extracellular blocking medium containing 20% rat serum (v/v) (Jackson, UK), 78% FACS buffer (v/v), and 2% (v/v) FC block (eBioscience) for 10 min and hereafter centrifuged. Then, cells were incubated with 25 µL antibody mixture (Table S1) for 30 min. Cells were washed twice with FACS‐EDTA buffer (5% (v/v) dFCS and 372 µg/mL EDTA in DPBS), and incubated with FACS lysing buffer (BD Biosciences) for 15 min to lyse red blood cells and fix the cells. Cells were again washed twice with the FACS‐EDTA buffer, and resuspended in 200 µL FACS‐EDTA buffer and stared at 4°C until analysis within 16 h.

### 2.4 Stimulation

Frozen spleen cells were used for the stimulation assays. Thawing was performed using a water bath and as soon as the suspension was minimally thawed an excess of RPMI was added. Hereafter cells were washed twice and counted with the Coulter Counter. 1 × 10^6^ cells were added to a 96‐well plate. For GD18, a PMA stimulation assay was performed and for GD10, both a PMA stimulation assay and an assay with irradiated male splenocytes were performed.

#### 2.4.1 PMA Stimulation

A stimulation mixture was added to the cells containing either 0.000048% (w/v) Phorbol 12‐Myristated 13‐Acetate (PMA), 0.0017% (w/v) Ionomycine, 0.0017% (w/v) 0.0002% Brefeldin A (all from Sigma‐Aldrich, USA) diluted in RPMI 10% dFCS buffer or a control mixture containing only 0.0002% (w/v) Brefeldin A and RPMI 10% dFCS was added. These mixtures were incubated at 37°C in a humified environment containing 5% CO_2_/95% O_2_ for 4 h.

#### 2.4.2 Irradiated Splenocytes Stimulation

To allow for specific stimulation with paternal antigens, male splenocytes from both C57BL6 and Balb/cJ mice were harvested, frozen, and thawed as described above. Male splenocytes were irradiated using 15 Gy with a dose rate 0.50 Gy/min (Cesium 137, IBL), to make sure the splenocytes themselves would be as less responsive as possibly, and used to stimulate the female splenocytes [21]. Irradiated splenocytes of C57BL6 and Balb/cJ were added in a 1:1 ratio to female Balb/cJ splenocytes that were either from a syngeneic pregnancy, an allogeneic pregnancy, or non‐pregnant females. These cells were incubated at 37°C in a humified environment containing 5% CO_2_/95% O_2_ for 24 h.

After stimulation with PMA or male splenocytes, antibody staining was performed as described above for the T cells, with the exception that a Zombie NIR solution (Biolegend, USA) was used for live/dead staining, and the extracellular and intracellular antibody mixes were as in Table S1.

### 2.5 Data Analysis and Statistics

The LSR‐ II flow cytometer system (BD Biosciences, USA) with FACS Diva software was used. Analysis was performed using Kaluza analysis version 2.1 software (Beckman Coulter, USA). The gating strategies are shown in Figures S1 and S2, unfortunately, gates were set and well characterized populations were used. Data were analyzed using Prism 8.0 (Graphpad Software Inc., USA). Outliers were removed using the ROUT method and data were checked for normality using the Kolmogorov–Smirnov test. If data were not normally distributed, data were log transformed. Fetal weight and placental weight were compared between syngeneic and semi‐allogeneic pregnancy using a student's *t* test. Litter size and number of resorptions were compared between syngeneic and semi‐allogeneic pregnancy using a Mann–Whitney *U* test. The subsets of T cells and monocytes were compared using a One‐way ANOVA, with Dunnet post‐testing. For the stimulation data a two‐way ANOVA was performed with Dunnet post‐testing. A *p* value < 0.05 was considered statistically significant; *p < 0.05, **p < 0.01, ***p < 0.001, ****p < 0.0001.

## 3 Results

### 3.1 Effect of Allogeneity on Fetal Weight, Placental Weight, Litter Size, and Number of Absorptions

**FIGURE 1:**
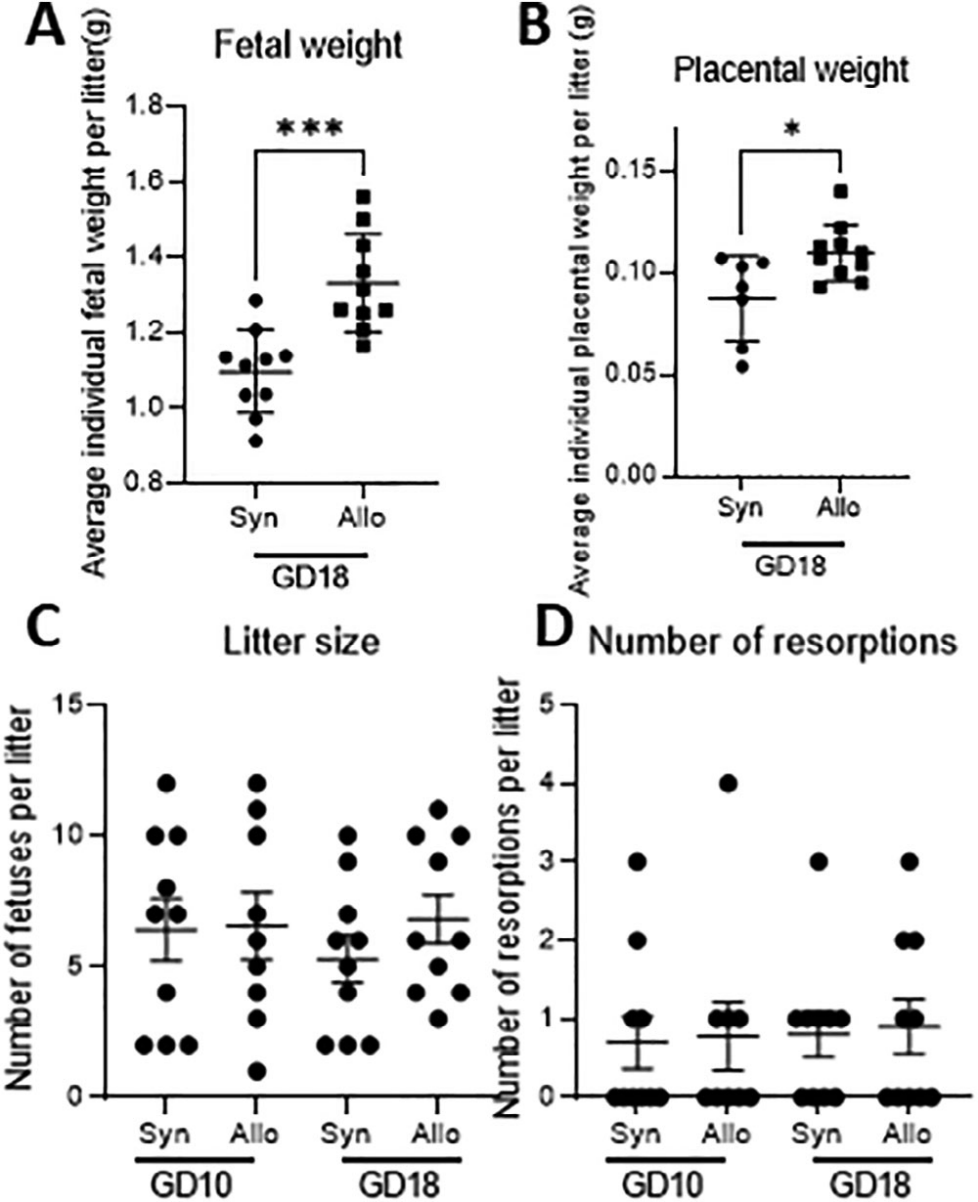
Average individual fetal and placental weight for the litters per mice in grams at GD18 (A, B), litter size and number of resorptions at GD10 and GD18 (C, D) for syngeneic and allogeneic mated mice. Each dot represents the mean weight of fetuses or placentas from a single litter (A, B). Statistical differences between syngeneic and semi‐allogeneic pregnant mice using student's *t*‐test (A, B), or a Mann–Whitney *U* test (C, D) (*p < 0.05; ***p < 0.001).

We first examined pregnancy outcomes in allogeneic and syngeneic mated mice. At GD18, allogeneic mated pregnant mice showed higher fetal and placental weights per litter than syngeneic mated pregnant mice did (*p* < 0.001; *p* < 0.05) (Figure 1A,B). Fetal and placental weights were not determined for GD10 due to the small size of individual fetuses and placentas. Litter size and the number of resorptions were not influenced by semi‐allogeneity, irrespective of GD (Figure 1C,D).

### 3.2 T Cell Subsets in the PALN and Spleen of Syngeneic and Semi‐Allogeneic Pregnancies

To determine the effects of semi‐allogeneity on maternal T cell subsets, we analyzed these within the PALN as a representative of the local immune system and the spleen as a representative of the peripheral immune system.

#### 3.2.1 CD3^+^ Cells, CD4^+^ T Cells, and CD8^+^ T Cells in the PALN

**FIGURE 2:**
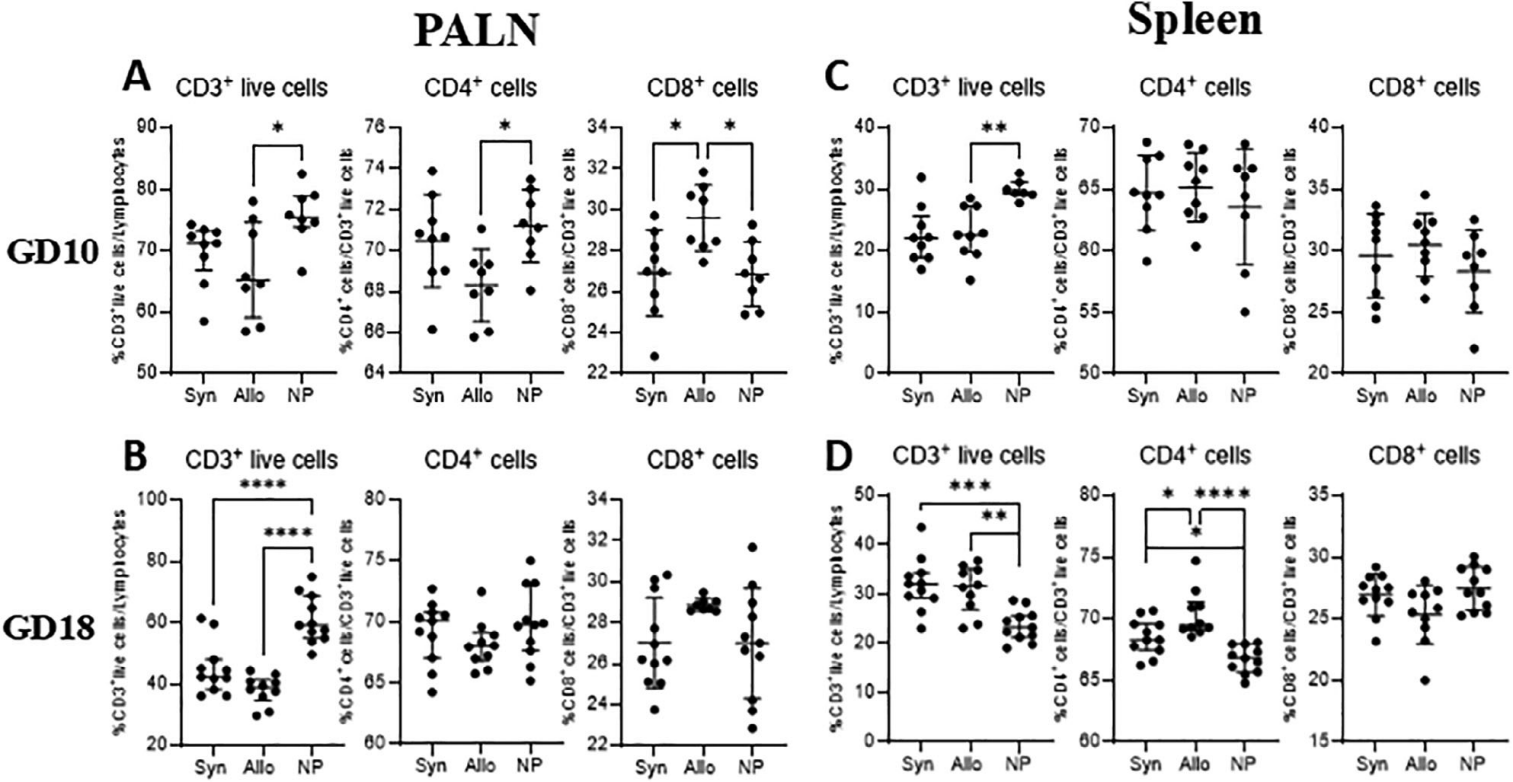
Differences in T cells from the PALN (A, B) (left) and spleen (C, D) (right), at GD10 (top row) and GD18 (lower row) comparing syngeneic, semi‐allogeneic, and non‐pregnant mice. Statistical differences between the groups were determined using a One‐way ANOVA, with Dunnet post‐testing (*p < 0.05; **p < 0.01; ***p < 0.001; ****p < 0.0001). allo = semi‐allogeneic pregnancy, Syn = syngeneic pregnancy, NP = non‐pregnant mice.

Figure 2A shows the percentage of CD3^+^ cells from total live cells, CD4^+^ T cells from CD3^+^ cells and CD8^+^ T cells from CD3^+^ cells, in the PALN at GD10 and GD18. At GD10, pregnant mice had a lower percentage of CD3^+^ cells from total live cells compared with non‐pregnant mice in the PALN, but this was only significant for allogeneic mated mice (*p* < 0.05). Furthermore, a lower percentage of CD4^+^ cells from CD3^+^ cells was found in allogeneic mated mice versus non‐pregnant mice (*p* < 0.05). A higher percentage of CD8^+^ cells from CD3^+^ cells was found in allogeneic mated mice compared to non‐pregnant mice and compared to syngeneic mated mice (*p* < 0.05). At GD18 (Figure 2B), the percentage of CD3^+^ cells from total live cells was lower in syngeneic mated mice and allogeneic mated mice compared to non‐pregnant mice (*p* < 0.0001). The percentage of CD4^+^ T cells from CD3^+^ cells and CD8^+^ T cells from CD3^+^ cells did not differ between the different groups of mice.

#### 3.2.2 CD3^+^ Cells, CD4^+^ T Cells, and CD8^+^ T Cells in the Spleen

At GD10, the percentage of CD3^+^ cells from total live cells in pregnant mice was lower than in non‐pregnant mice, though this was only significant for allogeneic mated mice (*p* < 0.01; see Figure 2C). No differences in percentage of CD4^+^ T cells from CD3^+^ cells and CD8^+^ T cells from CD3^+^ cells between the groups were found. At GD18, we found a higher percentage of CD3^+^ cells from total live cells in both groups of pregnant mice compared to non‐pregnant mice (*p* < 0.001 and *p* < 0.01, respectively; see Figure 2D). Additionally, the percentage of CD4^+^ T cells from CD3^+^ cells was greater in syngeneic and allogeneic mated mice than in non‐pregnant mice (*p* < 0.05 and *p* < 0.0001, respectively). Allogeneic mated mice also showed a higher percentage of CD4^+^ cells from CD3^+^ cells compared to syngeneic mated mice (*p* < 0.05). No differences between the three groups were found in percentage CD8^+^ cells from CD3^+^ cells.

#### 3.2.3 Memory T Cells in the PALN

**FIGURE 3:**
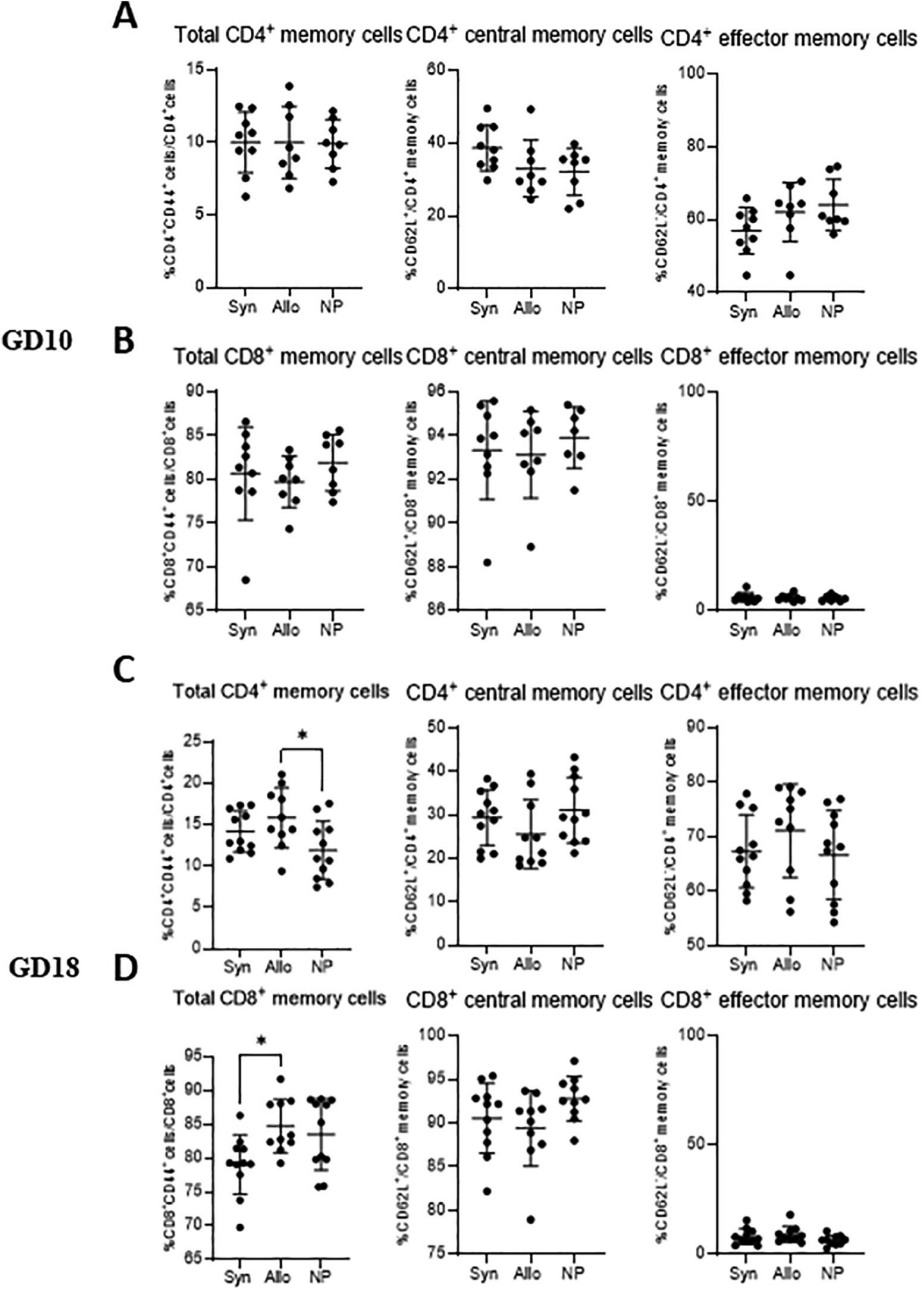
Differences in (subsets of) memory T cells from the PALN at GD10 (top two rows) and GD18 (lower two rows) comparing syngeneic, semi‐allogeneic, and non‐pregnant mice. Statistical differences between the groups were determined using a One‐way ANOVA, with Dunnet post‐testing (*p < 0.05). allo = allogeneic pregnancy, NP = non‐pregnant mice, Syn = syngeneic pregnancy.

We analyzed total as well as central and effector memory T cells in the PALN at both GD10 and GD18 (Figure 3). At GD10, there was no difference in (subsets of) memory T cells between the pregnant and non‐pregnant mice (Figure 3A,B). At GD18, the percentage of CD4^+^ memory cells from CD4^+^ cells were higher in allogeneic mated mice compared to non‐pregnant mice (*p* < 0.05) (Figure 3C). This difference was not observed in syngeneic mated mice, and there was no significant difference in CD4^+^ memory cells between pregnant mice groups. No differences between the groups were found for the analyzed CD4^+^ memory cell subsets (central and effector memory cells). The percentage of CD8^+^ memory cells from CD8^+^ cells were lower in syngeneic mated mice compared to allogeneic mated mice (*p* < 0.05) (Figure 3D). Yet, when comparing either group of pregnant mice to non‐pregnant mice, no significant difference in CD8^+^ memory cells was found. No differences between the groups were found for the CD8^+^ memory cell subsets (central and effector memory cells).

#### 3.2.4 Memory T Cells in the Spleen

**FIGURE 4:**
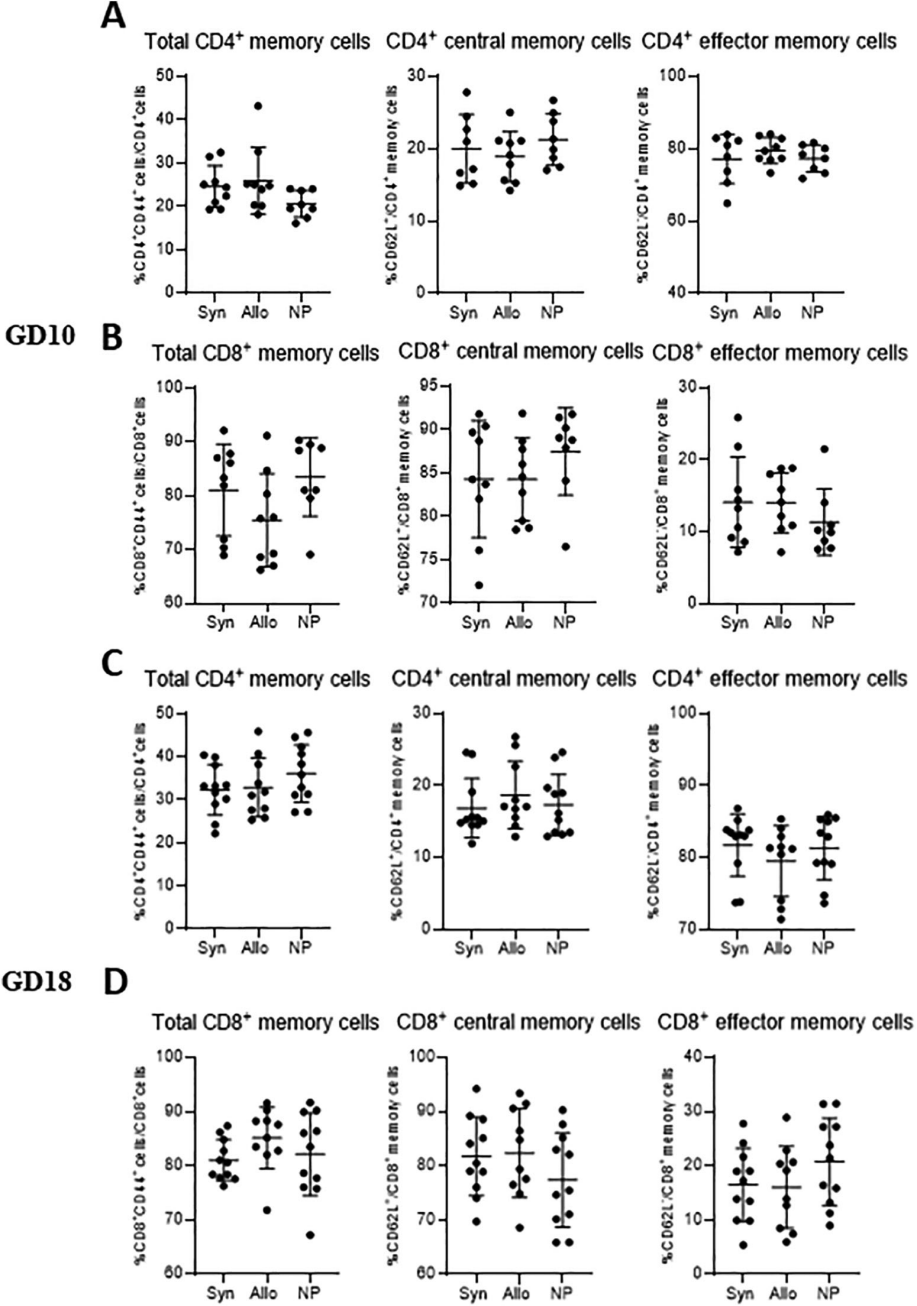
Differences in (subsets of) memory T cells from the spleen at GD10 (top two rows) and GD18 (lower two rows) comparing syngeneic, semi‐allogeneic, and non‐pregnant mice. Statistical differences between the groups were determined using a One‐way ANOVA, with Dunnet post‐testing (*p < 0.05). allo = allogeneic pregnancy, NP = non‐pregnant mice, Syn = syngeneic pregnancy.

At GD10 and GD18, we observed no difference in memory T cell subsets in the spleen (Figure 4A–D), between the pregnant and non‐pregnant mice.

#### 3.2.5 Treg Cells and Memory Treg Cells in the PALN and Spleen

**FIGURE 5:**
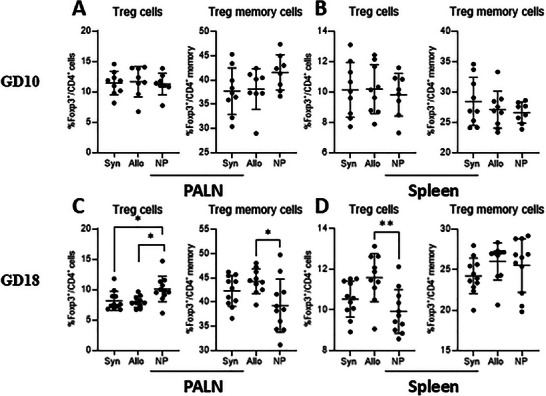
Differences in Tregs and memory Tregs from the PALN (left) and spleen (right) at GD10 (top row) and GD18 (lower row) comparing syngeneic, semi‐allogeneic, and non‐pregnant mice. Statistical differences between the groups were determined using a One‐way ANOVA, with Dunnet post‐testing (*p < 0.05; **p < 0.01). allo = semi‐allogeneic pregnancy, NP = non‐pregnant mice, Syn = syngeneic pregnancy, Treg = regulatory T cell.

At GD10 we observed no difference in the percentages of Treg cells from CD4^+^ cells and Treg memory cells from CD4^+^ memory cells, among the groups of mice in both the PALN and spleen (Figure 5A,B). In the PALN at GD18 (Figure 5C), both groups of pregnant mice had a lower percentage of Treg memory cells from CD4^+^ memory cells than non‐pregnant mice (*p* < 0.05 for both). Treg cells did not differ between syngeneic mated and allogeneic mated mice. The percentage of Treg memory cells from CD4^+^ memory cells were higher in allogeneic mated mice compared to non‐pregnant mice in the PALN (*p* < 0.05). The percentage of Treg cells from CD4^+^ T cells, from the spleen at GD18 were higher in allogeneic mated mice (*p* < 0.01) compared to non‐pregnant mice (Figure 5D). Treg memory cells did not differ between the groups of mice.

### 3.3 IFNγ and IL4 Production From T Cells After Stimulation With PMA or Paternal Antigens

#### 3.3.1 IFNγ and IL4 Production in Splenic T Cells After Stimulation With PMA

**FIGURE 6:**
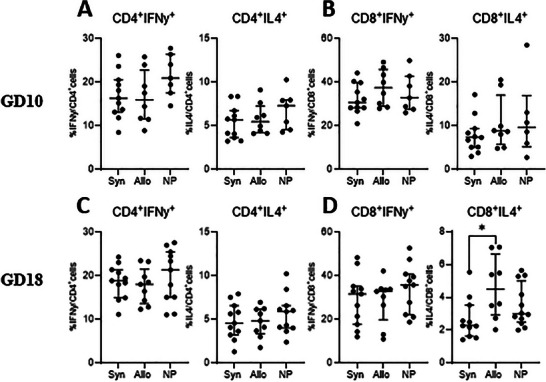
Differences after T cell stimulation with PMA at GD10 (upper row) and GD18 (lower row) comparing syngeneic, semi‐allogeneic, and non‐pregnant mice. Statistical differences between the groups were determined using a One‐way ANOVA, with Dunnet post‐testing (*p < 0.05). allo = allogeneic pregnancy, NP = non‐pregnant mice, Syn = syngeneic pregnancy.

To assess the production of IFNγ and IL4 by CD4^+^ and CD8^+^ T cells, we performed PMA stimulation assays on splenocytes isolated at GD10 and GD18 (Figure 6A–D). At GD10, the percentages of CD4^+^IFNγ^+^ and CD4^+^IL4^+^ cells from CD4^+^ T cells were similar in syngeneic, semi‐allogeneic, and non‐pregnant mice (Figure 6A). Likewise, there was no difference in the production of IFNγ and IL4 by CD8^+^ T cells after PMA stimulation (Figure 6B). By GD18, the production of IFNγ and IL4 by CD4^+^ T cells remained unchanged across the groups. However, syngeneic mated mice had a lower percentage of CD8^+^IL4^+^ cells from CD8^+^ T cells compared to allogeneic mated mice (Figure 6D).

#### 3.3.2 IFNγ and IL4 Production in Splenic T Cells After Stimulation With Paternal Antigens

**FIGURE 7:**
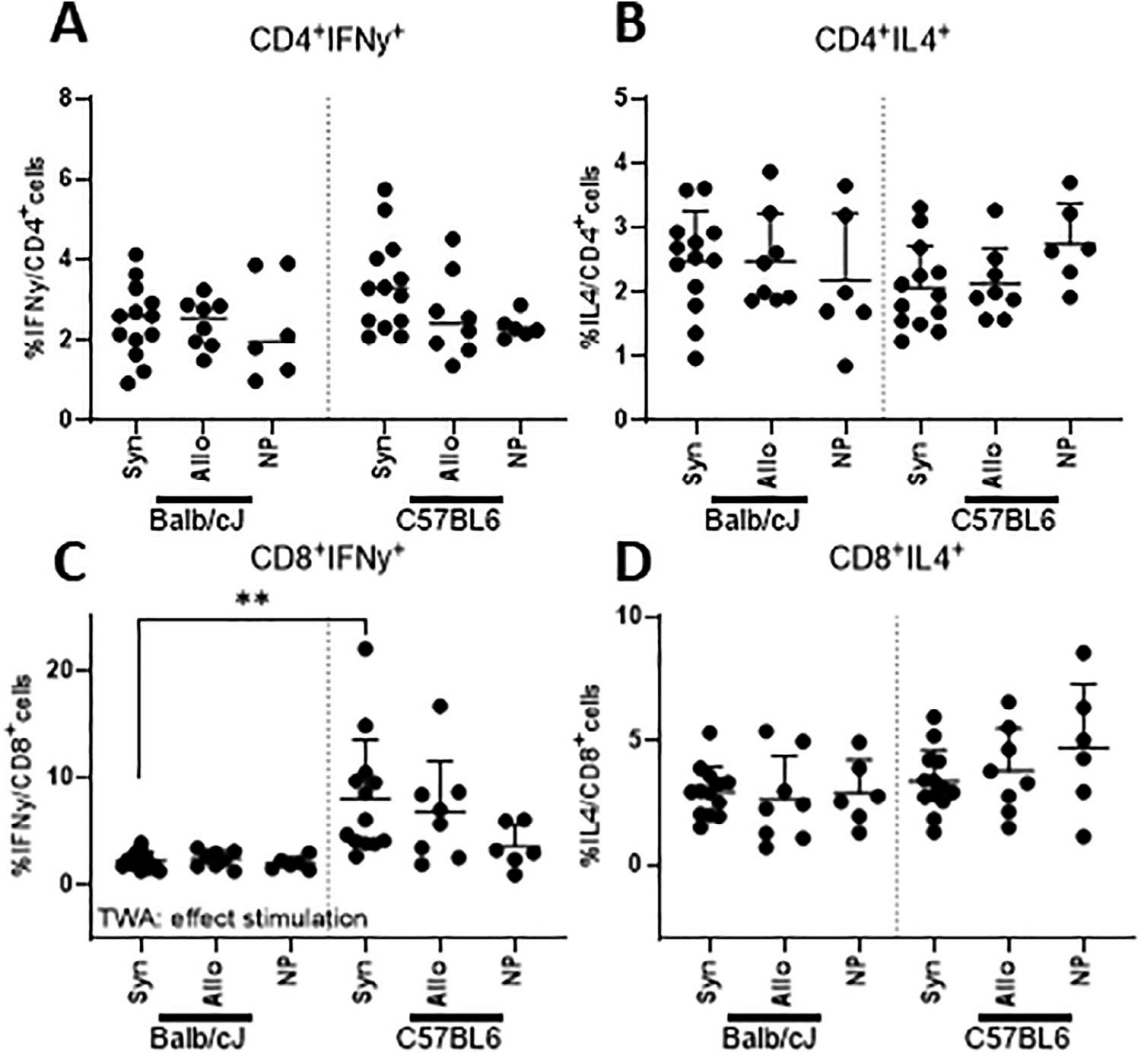
Differences after T cell stimulation with either Balb/cJ irradiated lymphocytes or C57BL6 irradiated splenocytes at GD10, comparing syngeneic, allogeneic, and non‐pregnant mice. Statistical differences between the groups were determined using a Two‐way ANOVA (TWA), with Dunnet post‐testing (*p < 0.05; **p < 0.01). allo = allogeneic pregnancy, NP = non‐pregnant mice, Syn = syngeneic pregnancy.

To determine the responsiveness of CD4^+^ and CD8^+^ T cells to paternal antigens, we used a paternal antigen stimulation assay. We stimulated splenocytes from syngeneic (Balb/cJ × Balb/cJ) and allogeneic (Balb/cJ × C57BL6) mated pregnant mice as well as from non‐pregnant mice (Balb/cJ) with splenocytes from either Balb/cJ or C57BL6 male mice. Using a Two‐way ANOVA, for CD4^+^ T cells, no effects of allogeneity or stimulation were found in the percentage of cells producing IFNγ or IL4 from CD4^+^ T cells after stimulation with either Balb/cJ or C57Bl6 male mice (see Figure 7A,B). For CD8^+^ cells, stimulation with C57BL6 male splenocytes led to a higher percentage of IFNγ producing cells from CD8^+^ T cells compared to stimulation with Balb/cJ male splenocytes, but this effect was only significant in syngeneic mated mice stimulated with C57BL6 male splenocytes (*p* < 0.01) (Figure 7C).

### 3.4 Blood Leukocyte Subsets, Monocytes Subsets, and Monocyte Activation Status

#### 3.4.1 Leukocyte Subsets

**FIGURE 8:**
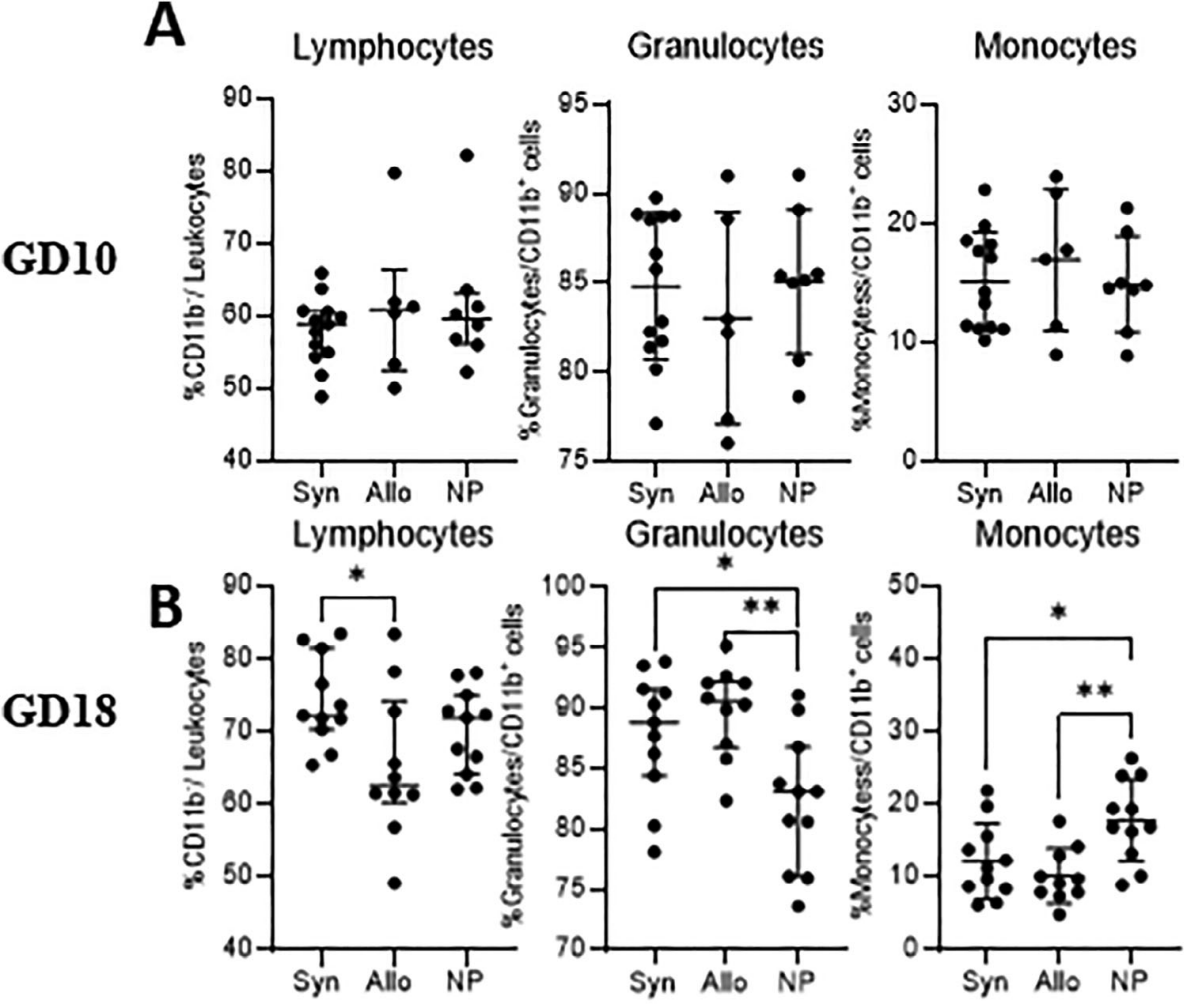
Differences in lymphocytes, granulocytes and monocytes from blood at GD10 (top row) and GD18 (lower row) comparing syngeneic, semi‐allogeneic, and non‐pregnant mice. Statistical differences between the groups were determined using a One‐way ANOVA, with Dunnet post‐testing. allo = semi‐allogeneic pregnancy, NP = non‐pregnant mice, Syn = syngeneic pregnancy.

At GD10, there were no differences in the percentages of lymphocytes from the total leukocytes, granulocytes and monocytes (from the total CD11b^+^ population) in the peripheral blood between the groups of mice (Figure 8A). At GD18 (Figure 8B), syngeneic mated mice had a higher percentage of lymphocytes from the total leukocyte population than allogeneic mated mice (*p* < 0.05). Both groups of pregnant mice showed higher percentages of granulocytes from the total CD11b^+^ population compared to non‐pregnant mice (*p* < 0.05 and *p* < 0.01, respectively). Monocyte percentages from the total CD11b^+^ population were lower in both pregnant mouse groups in comparison to non‐pregnant mice (*p* < 0.05 and *p* < 0.01, respectively).

#### 3.4.2 Monocyte Subsets

**FIGURE 9:**
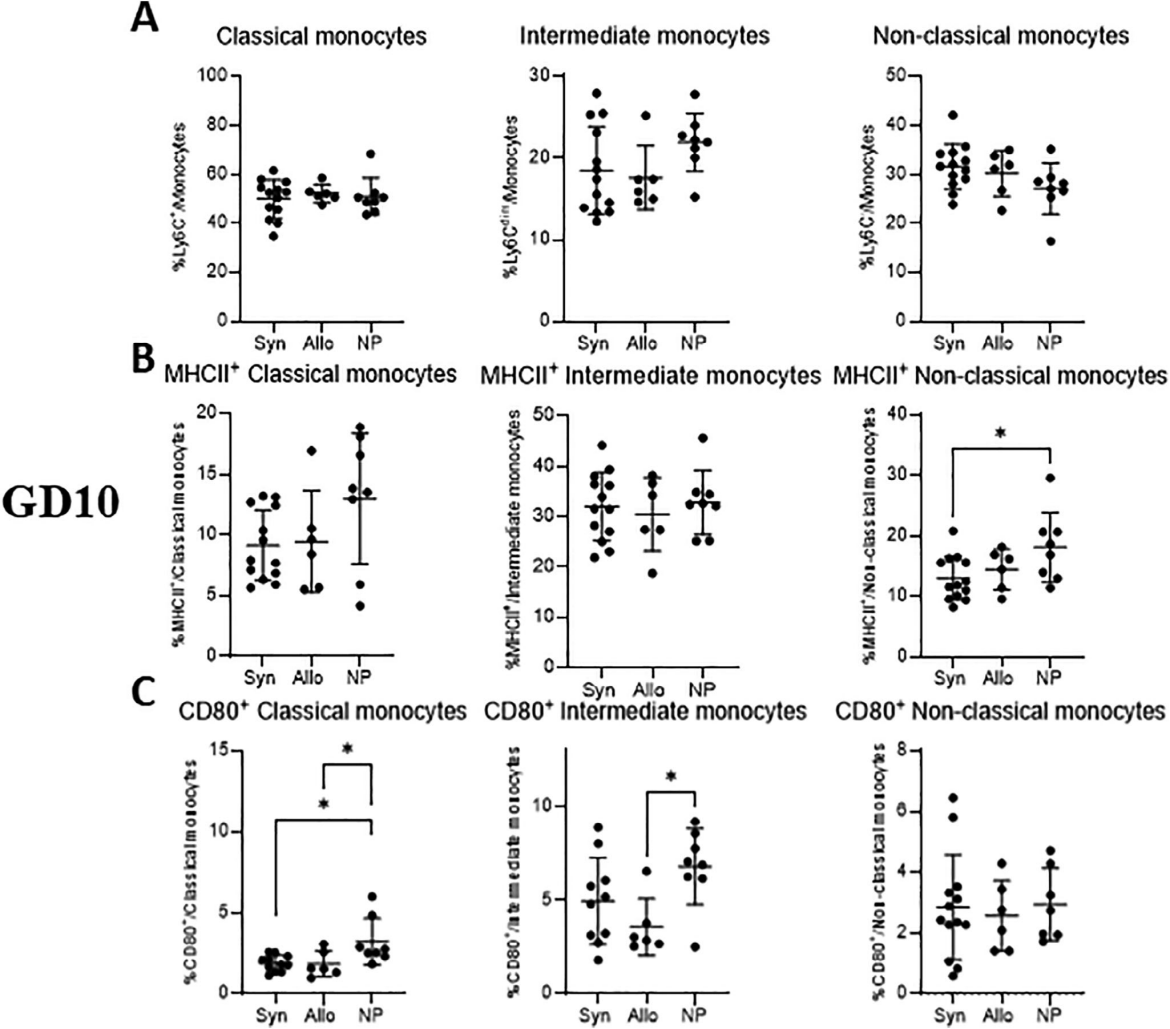
Differences in monocyte subsets and functional markers (MHCII and CD80) of monocytes from blood at GD10 comparing syngeneic, semi‐allogeneic, and non‐pregnant mice. Statistical differences between the groups were determined using a One‐way ANOVA, with Dunnet post‐testing (*p < 0.05; **p < 0.01; ***p < 0.001; ****p < 0.0001). allo = semi‐allogeneic pregnancy, NP = non‐pregnant mice, Syn = syngeneic pregnancy.

**FIGURE 10:**
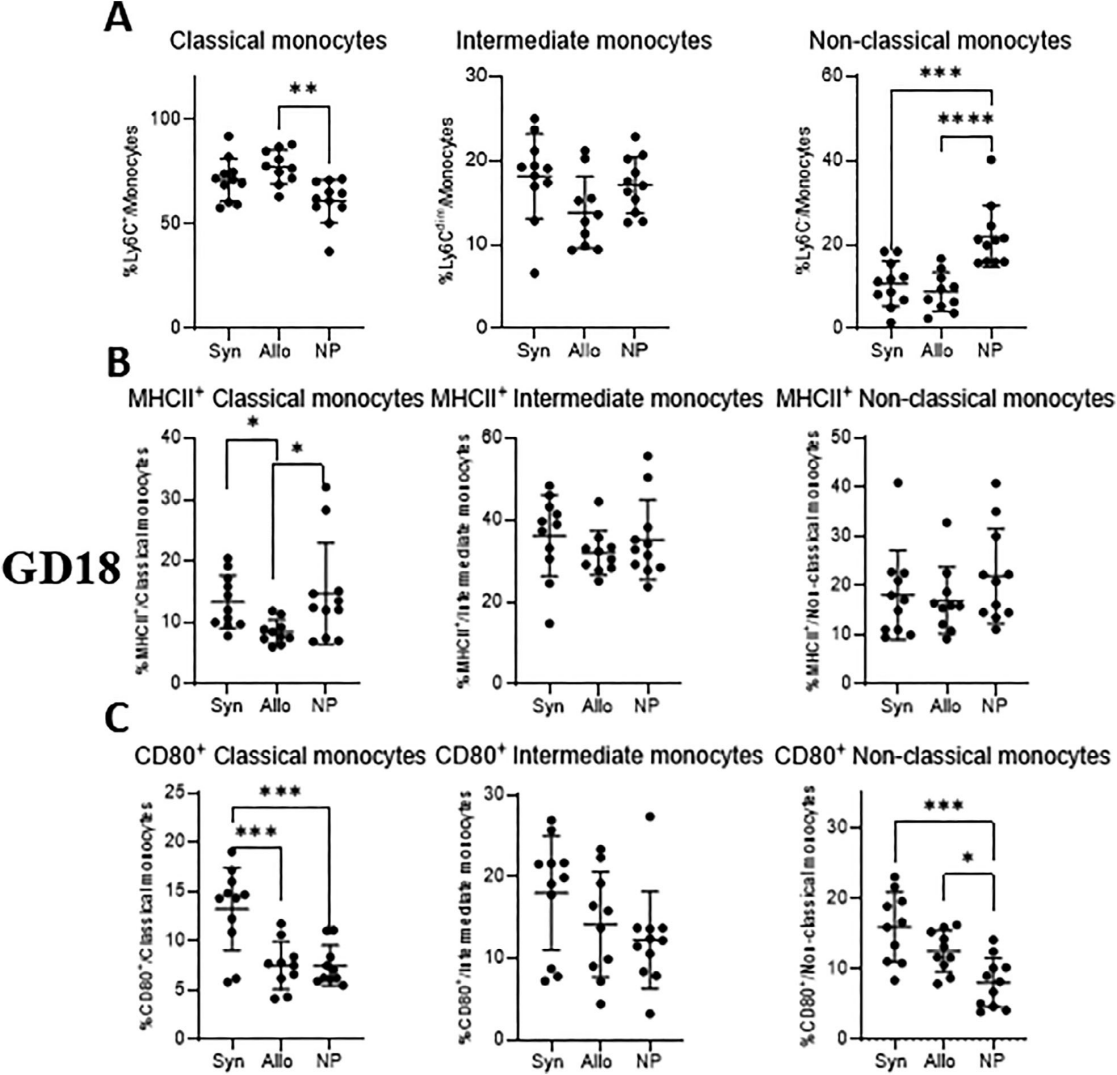
Differences in monocyte subsets and functional markers (MHCII and CD80) of monocytes from blood at GD18 comparing syngeneic, semi‐allogeneic, and non‐pregnant mice. Statistical differences between the groups were determined using a One‐way ANOVA, with Dunnet post‐testing (*p < 0.05; **p < 0.01; ***p < 0.001; ****p < 0.0001). allo = semi‐allogeneic pregnancy, NP = non‐pregnant mice, Syn = syngeneic pregnancy.

At GD10, there were no differences among the groups for the three monocyte subsets: classical, intermediate and non‐classical (Figure 9). At GD18 (Figure 10), Both groups of mice showed an increased percentage of classical monocytes from the total monocytes, compared to non‐pregnant mice, although this was only significant for allogeneic mated mice (*p* < 0.01) (Figure 10A). No differences between the three groups were found in intermediate monocytes. Both groups of pregnant mice had lower percentages of non‐classical monocytes from the total monocytes compared to non‐pregnant mice (*p* < 0.001 and *p* < 0.0001, respectively) (Figure 10C).

#### 3.4.3 Monocyte Activation Status

To assess activation of monocyte subsets, we used MHCII and CD80 (Figures 9 and 10). At GD10, no differences in MHCII expression were observed for classical and intermediate monocytes across the three groups (Figure 9). For non‐classical monocytes, pregnant mice displayed a lower percentage of MHCII^+^ from the total non‐classical monocytes, compared to non‐pregnant mice (*p* < 0.05), but this was only statistically significant for syngeneic mated mice (Figure 9B). In terms of CD80 expression, classical monocytes from both syngeneic and allogeneic mated mice showed a lower percentage of CD80^+^ from classical monocytes compared to non‐pregnant mice (*p* < 0.05) (Figure 9C). For intermediate monocytes, a lower percentage of CD80^+^ from intermediate monocytes was only observed in allogeneic mated pregnant mice compared to non‐pregnant mice (*p* < 0.05) (Figure 9C). There were no differences in CD80^+^ expression on non‐classical monocytes across the groups.

At GD18, allogeneic mated mice had a lower percentage of MHCII^+^ classical monocytes from classical monocytes compared to syngeneic mated mice and non‐pregnant mice (*p* < 0.05 for both) (Figure 10B). For intermediate and non‐classical monocytes, there were no differences between the groups in the percentage of cells expressing MHCII. Syngeneic mated mice had a higher percentage of CD80^+^ classical monocytes from classical monocytes than both allogeneic mated and non‐pregnant mice (*p* < 0.001, for both), there were no differences in CD80 expression among intermediate monocytes across the three groups (Figure 10C). Both groups of pregnant mice had a higher percentage of CD80^+^ non‐classical monocytes from non‐classical monocytes compared to non‐pregnant mice (*p* < 0.001, *p* < 0.05, respectively) (Figure 10C).

## 4 Discussion

The role of allogeneic fetal tissue in inducing maternal immune adaptations in mid and late pregnancy is relatively unknown. In this study, we examined the effects of fetal allo‐antigens on maternal immune cells by comparing immunological changes during mid and late pregnancy in syngeneic and allogeneic mated mice. Interestingly, most maternal immune cells and subsets were found to be similar in syngeneic and allogeneic mated pregnancies. In our study we observed only minor differences in percentages of CD3, CD4, CD8, and Treg cells in the PALN and spleens, and memory T cell subsets in the PALN, between syngeneic and ‐allogeneic mated pregnant mice. Although the changes in monocyte subsets were similar in both types of pregnancies, the activation status of the monocyte subsets, as indicated by CD80 and MHCII expression varied between allogeneic and syngeneic mated mice.

At GD18, our results showed that both fetal and placental weights in allogeneic mated pregnant mice were higher compared to that of syngeneic mated mice. This could suggest a better placental and fetal development in allogeneic mated mice. A possible explanation for this could be different recognition of paternal antigens by trophoblasts [22]. A possible confounding factor could be an effect of the different mouse strains we used. Balb/cJ females were mated with Balb/cJ male mice for syngeneic pregnancies and with C57BL/6 male for semi‐allogeneic pregnancies, strain specific characteristics might explain higher fetal weights. However, in a recent study, we used pregnant syngeneic C57BL/6 mice and fetal placental weight from these mice were comparable with the syngeneic pregnant Balb/cJ mice in the present study, suggesting that the difference in fetal and placental weight is not an effect of strain difference [18].

Previous work from our group found differences in memory T cells in human pregnancy, with a higher percentage CD4^+^ memory cells (mainly effector memory cells) in pregnant compared with non‐pregnant women [23]. In addition, mice studies have shown alterations in memory T cell subsets during pregnancy [10, 24, 25]. In our study, we analyzed the effects of allo‐antigens on memory T cell subsets. We found slight differences in memory T cell subsets between pregnant and non‐pregnant mice in the PALN at day 18, while the percentage of total CD4^+^ and CD8^+^ memory cells was higher in allogenic mated mice compared with non‐pregnant mice or syngeneic mated mice respectively. In the PALN these effects of allogeneity were not observed in the subpopulations of CD4^+^ effector memory T cells and CD4^+^ central memory T cells. Whether the increased CD4^+^ and CD8^+^ memory T cells are paternal antigen specific memory T cells needs to be determined.

To further differentiate the immune response of memory T cells between allogeneic mated mice and syngeneic mated mice, we used stimulation experiments. Due to the limited number of leukocytes from the PALN, the stimulation experiments were only performed on splenocytes. In the first stimulation experiment, we stimulated splenocytes with PMA and ionomycin and evaluated the expression of IFNγ and IL4. We did not see significant differences in IFNγ or IL4 production between splenocytes from syngeneic mated mice and splenocytes from allogeneic mated mice, although at both day 10 and day 18, IFNγ production was lower in splenocytes of pregnant mice versus non‐pregnant mice. Previous studies from our lab found a decreased Th1 response in pregnant versus non‐pregnant mice at ay 18 of pregnancy [18]. Differences in methods used (stimulation of splenocytes vs. staining with Th1 transcription factor Tbet) may explain the difference. At GD18 a higher percentage of CD8^+^IL4^+^ cells in allogeneic mated mice compared to syngeneic mated mice was found. Considering that IL4 is predominantly associated with CD4^+^ T cells in immune regulation, the significance of this finding from CD8^+^ T cells remains unclear [26].

In the second stimulation experiment, we used splenocytes from either Balb/cJ or C57BL6 mice to stimulate the maternal splenocytes. Using this method, we aimed at finding paternally specific memory cells in allogeneic mated mice, we expected that these paternal specific cells would respond stronger to stimulation by either BALB/cJ (syngeneic) or C57BL6 mice (semi‐allogeneic). Interestingly, we only found a difference in stimulation with C57BL6 splenocytes in the production of IFNγ by CD8^+^ memory T cells as compared with stimulation with syngeneic splenocytes (i.e., splenocytes from Balb/cJ mice). As said before, this may suggest that there are no or only few paternally specific memory T cells in the spleen at day 10 of pregnancy. Locally at the PALN these effects could be stronger. This observation also suggest that pregnancy factors other than paternally specific factors, such as genetic disparities between these mouse strains, may be responsible for this heightened response of Balb/cJ pregnant mice to C57BL6 mice. Genetic differences could potentially influence the recognition and immune response of CD8^+^ memory T cells (BALBC/J) to C57BL6 splenocytes.

Tregs are considered important modulators of an semi‐allogeneic pregnancy [10, 27–29], and are known for (amongst others) producing IL10 and TGFβ [20]. Previous research showed that in a semi‐allogeneic pregnancy, the absence of Tregs in early pregnancy leads to adverse pregnancy outcomes, while in syngeneic pregnancy absence of Tregs does not result in adverse pregnancy outcome [30]. In our study, we observed an effect of pregnancy itself on Treg cells on day 18 in the PALN, that is, decreased Treg in pregnant mice, rather than a distinct effect of allogeneity. An effect of allogeneity was present at day 18 in the spleen, where Tregs were increased only in allogeneic mated mice only. Notably, we only evaluated Treg cells in mid‐pregnancy and late pregnancy. As it has been shown that Treg dynamics change throughout pregnancy, more prominent pregnancy or semi‐allogeneic effects may have been visible in early pregnancy or between days 10 and 18 [30]. When analyzing Treg memory cells, our results indicate minimal differences, with the exception of GD18 in the PALN, where allogeneic mate pregnancies exhibited a higher presence of Treg memory cells compared to non‐pregnant mice (but not syngeneic pregnancies). This may be in line with observation by Rowe et al. [10], who found accumulation of memory Treg during semi‐allogeneic pregnancy. This accumulation was faster, with better pregnancy outcomes in a second semi‐allogeneic pregnancy.

Not only T cells, but also innate immune cells are known to change during pregnancy [31, 32]. Most studies found a proinflammatory condition during pregnancy, with activation of monocytes [32]. We observed a decreased percentage of monocytes in the peripheral blood of pregnant mice regardless whether the pregnancy was syngeneic or semi‐allogeneic, toward the end of pregnancy (GD18), when compared to non‐pregnant mice. This suggests that these effects are primarily induced by pregnancy itself and are not dependent on the presence of allogeneic fetal tissue. In our study, subsets of monocytes and their activation status were predominantly influenced by the effect of pregnancy rather than allogeneity. At day 18 of pregnancy, we found higher percentages of classical monocytes and higher percentages of non‐classical monocytes in pregnant mice. This is in line with previous studies from our lab [18]. However, with respect to the activation status, there were some effects of the presence of the semi‐allogeneic fetus. This suggests that the presence of an allogeneic fetus likely affects monocyte activation. The circulating monocytes may become more activated due to their circulation through the placenta, during which they may encounter the semi‐allogeneic trophoblast.

In conclusion, our study highlights that immune adaptations ad mid and late pregnancy are likely largely driven by pregnancy itself, with less changes driven by fetal semi‐allogeneity. This study enhances our comprehension of the regulation of the changes of the immune system that take place during pregnancy.

## Ethics Statement

The authors confirm that the ethical policies of the journal, as noted on the journal's author guidelines page, have been adhered to, and the appropriate ethical review committee approval has been received. This animal study protocol was approved by the Central Committee for Animal Experimentation in the Netherlands (AVD 1050020185904).

## Conflicts of Interest

The authors declare no conflicts of interest.

## Supporting information




**Figure S1:** Gating strategy for T cell staining. First, lymphocytes were selected from the forward/sideward (FSC/SSC) scatterplot of all events (Suppl. Figure 1A).Next, Live CD3^+^ cells were identified (Suppl. Figure 1B), from these cells CD4^+^ and CD8^+^ cells were selected (Suppl. Figure 1C), and from here using CD44, the memory T cells selected for both the CD4^+^ as well as the CD8^+^ T cells (Suppl. Figure 1D, F). To identify the effector as well as the central memory cells CD62L was used (Suppl. Figure 1E,G). Activation was for all subsets determined using CD69, in this figure an example is given for the CD4^+^ memory cells (Suppl. Figure 1H). From the CD4^+^ cells and CD4^+^ memory cells, Treg cells and Treg memory cells were selected, using FoxP3 (Suppl. Figure 1I,J). In the stimulation experiment, the CD4^+^ and CD8^+^ T cells were identified as described above and shown here again (Suppl. Figure 1K‐M). Using IFNg as well as IL4 the activation of CD4^+^ T cells and CD8^+^ T cells were determined (Suppl. Figure 1N‐Q).
**Figure S2:** Gating strategy for the monocyte staining; First, from the FSC/SSC scatterplot, leukocytes were selected (and dead cells excluded) (Suppl. Figure 2A). Next, CD11b^+^, CD43^+^ cells were selected (Suppl. Figure 2B), and from here on monocytes and granulocytes were identified (Suppl. Figure 2C). Using Ly6C the different subsets of monocytes were selected (Suppl. Figure 2D). Next, using the activation markers MHCII^+^ and CD80^+^, activation status was determined for all three subsets (here only shown for classical monocytes) (Suppl. Figure 2E,F).
**Table S1:** Extracellular and intracellular antibody mixes.

## Data Availability

The data that support the findings of this study are available on request from the corresponding author. The data are not publicly available due to privacy or ethical restrictions.
