# Supplementary material for: Maternal Immune Characteristics at Mid and Late Pregnancy Are Mostly Independent of Fetal Allogeneity in Mice
Source: Am J Reprod Immunol. 2025 Jun 1;93(6):e70089. doi: 10.1111/aji.70089 (PMC12127012; doi:10.1111/aji.70089)
Supplement: Supplementary file 1 — Figure S1: Gating strategy for T cell staining. First, lymphocytes were selected from the forward/sideward (FSC/SSC) scatterplot of all events (Suppl. Figure 1A).Next, Live CD3+ cells were identified (Suppl. Figure 1B), from these cells CD4+ and CD8+ cells were selected (Suppl. Figure 1C), and from here using CD44, the memory T cells selected for both the CD4+ as well as the CD8+ T cells (Suppl. Figure 1D, F). To identify the effector as well as the central memory cells CD62L was used (Suppl. Figure 1E,G). Activation was for all subsets determined using CD69, in this figure an example is given for the CD4+ memory cells (Suppl. Figure 1H). From the CD4+ cells and CD4+ memory cells, Treg cells and Treg memory cells were selected, using FoxP3 (Suppl. Figure 1I,J). In the stimulation experiment, the CD4+ and CD8+ T cells were identified as described above and shown here again (Suppl. Figure 1K‐M). Using IFNg as well as IL4 the activation of CD4+ T cells and CD8+ T cells were determined (Suppl. Figure 1N‐Q). Figure S2: Gating strategy for the monocyte staining; First, from the FSC/SSC scatterplot, leukocytes were selected (and dead cells excluded) (Suppl. Figure 2A). Next, CD11b+, CD43+ cells were selected (Suppl. Figure 2B), and from here on monocytes and granulocytes were identified (Suppl. Figure 2C). Using Ly6C the different subsets of monocytes were selected (Suppl. Figure 2D). Next, using the activation markers MHCII+ and CD80+, activation status was determined for all three subsets (here only shown for classical monocytes) (Suppl. Figure 2E,F). Table S1: Extracellular and intracellular antibody mixes. [file AJI-93-e70089-s001.docx]

**Supplementary data**

| **Supplementary Table 1.** Extracellular and intracellular antibody mixes | | | | | | |
| --- | --- | --- | --- | --- | --- | --- |
| **Marker** | **Fluorochrome** | **Clone** | **Panel** | **Antibody mix** | **Dilution** | **Company** |
| CD43 | APC | S11 | Monocytes | Extracellular | 100x | BioLegend |
| CD115 | PE-Cy7 | AFS98 | Monocytes | Extracellular | 60x | BioLegend |
| CD11b | PE | M1/70 | Monocytes | Extracellular | 200x | BioLegend |
| Ly6C | AF488 | HK1.4 | Monocytes | Extracellular | 200x | BioLegend |
| Ly6G | BV605 | 1A8 | Monocytes | Extracellular | 25x | BD |
| CD80 | BV421 | 16-10A1 | Monocytes | Extracellular | 75x | BioLegend |
| MHCII | PerCp-Cy5.5 | M5/114.14.2 | Monocytes | Extracellular | 200x | BioLegend |
| CD3 | BV421 | 17A2 | T cells | Extracellular | 50x | BioLegend |
| CD4 | PerCp-Cy5.5 | GK1.5 | T cells | Extracellular | 100x | BioLegend |
| CD8 | APC-Cy7 | 53-6.7 | T cells | Extracellular | 50x | BioLegend |
| CD44 | BUV737 | IM7 | T cells | Extracellular | 50x | BD |
| CD62L | BUV395 | MEL-14 | T cells | Extracellular | 50x | BD |
| FoxP3 | FITC | FJK-16S | T cells | Intracellular | 50x | ThermoFisher |
| CD3 | BV605 | 17A2 | Stimulation | Extracellular | 25x | BioLegend |
| CD4 | PE-Cy7 | GK1.5 | Stimulation | Extracellular | 100x | BioLegend |
| CD8 | PerCP-Cy5.5 | 53-6.7 | Stimulation | Extracellular | 50x | BioLegend |
| IFNg | PE | XMG1.2 | Stimulation | Intracellular | 500x | BioLegend |
| IL4 | FITC | BVD6-24G2 | Stimulation | Intracellular | 400x | ThermoFisher |
| IL10 | BV421 | JES5-16E3 | Stimulation | Intracellular | 300x | BioLegend |
| IL17a | APC | TC11-18H10.1 | Stimulation | Intracellular | 500x | BioLegend |
|  |  |  |  |  |  |  |

**Supplementary Figures**

**CD8^+^ central memory**

**Lymphocytes**

SSC-A

FSC-A

CD3

Live/dead

**CD3^+^ live cells**

**CD3^+^ live cells**

**CD4^+^**

**CD8^+^**

CD4

CD8

**CD4^+^**

CD4

CD44

**CD4^+^ memory**

**CD4^+^ effector memory**

**CD4^+^ central memory**

**CD8^+^**

CD4

CD62L

CD8

CD44

**CD8^+^ memory**

**CD8^+^ memory**

**CD8^+^ effector memory**

**CD4^+^ memory**

CD4

CD69

**Activated CD4^+^ memory**

**A**

CD8

CD62L

**CD4^+^ memory**

**B**

**C**

**D**

**E**

**F**

**G**

**H**

**Lymphocytes**

**Lymphocytes**

SSC-A

FSC-A

Live/Dead

CD3

**CD3^+^ live cells**

**CD3^+^ live cells**

**CD4^+^**

**CD8^+^**

CD4

CD8

CD4

CD4

IFNg

IFNg

IL4

IL4

CD8

CD8

**CD4^+^**

**CD4^+^**

**CD8^+^**

**CD8^+^**

**CD4^+^IFNg^+^**

**CD8^+^IFNg^+^**

**CD4^+^IL4^+^**

**CD8^+^IL4^+^**

**K**

**L**

**M**

**N**

**O**

**P**

**Q**

**CD4^+^**

CD4

FoxP3

**Treg**

**CD4^+^ memory**

CD4

FoxP3

**Treg memory**

**I**

**J**

**Supplementary figure 1. Gating strategy for T cell staining.** First, lymphocytes were selected from the forward/sideward (FSC/SSC) scatterplot of all events (Suppl. Figure 1A).Next, Live CD3^+^ cells were identified (Suppl. Figure 1B), from these cells CD4^+^ and CD8^+^ cells were selected (Suppl. Figure 1C), and from here using CD44, the memory T cells selected for both the CD4^+^ as well as the CD8^+^ T cells (Suppl. Figure 1D, F). To identify the effector as well as the central memory cells CD62L was used (Suppl. Figure 1E,G). Activation was for all subsets determined using CD69, in this figure an example is given for the CD4^+^ memory cells (Suppl. Figure 1H). From the CD4^+^ cells and CD4^+^ memory cells, Treg cells and Treg memory cells were selected, using FoxP3 (Suppl. Figure 1I,J). In the stimulation experiment, the CD4^+^ and CD8^+^ T cells were identified as described above and shown here again (Suppl. Figure 1K-M). Using IFNg as well as IL4 the activation of CD4^+^ T cells and CD8^+^ T cells were determined (Suppl. Figure 1N-Q) .

SSC-A

FSC-A

CD11b

CD43

**Leukocytes**

**Leukocytes**

**CD11b^+^**

**CD11b^+^**

**Granulocytes**

**Monocytes**

Ly6G

CD115

**Monocytes**

**Non-classical**

**Intermediate**

**Classical**

CD43

Ly6C

**Classical**

MHCII

CD80

CD11b

CD11b

**Classical**

**A**

**MHCII^+^**

**CD80^+^**

**B**

**C**

**D**

**E**

**F**

**Supplementary figure 2. Gating strategy for the monocyte staining**; First, from the FSC/SSC scatterplot, leukocytes were selected (and dead cells excluded) (Suppl. Figure 2A). Next, CD11b^+^, CD43^+^ cells were selected (Suppl. Figure 2B), and from here on monocytes and granulocytes were identified (Suppl. Figure 2C). Using Ly6C the different subsets of monocytes were selected (Suppl. Figure 2D). Next, using the activation markers MHCII^+^ and CD80^+^, activation status was determined for all three subsets (here only shown for classical monocytes) (Suppl. Figure 2E,F).
